# Optimization of Growth Conditions of *Lentinus edodes* Mycelium and Polysaccharides on Walnut Shell by-Products Using Response Surface Analysis

**Published:** 2018

**Authors:** Mahdieh Ameri Shah Reza, Hossein Vahidi, Farzad Kobarfard

**Affiliations:** a *Department of Pharmaceutical Biotechnology, School of Pharmacy, Shahid Beheshti University of Medical Sciences, Tehran, Iran. *; b *Student’s Research Committee, Shahid Beheshti University of Medical Sciences, Tehran, Iran.*; c *Department of Medicinal Chemistry, School of Pharmacy, Shahid Beheshti University of Medical Sciences, Tehran, Iran.*

**Keywords:** Biomass, Polysaccharides, *Lentinus edodes*, Solid- state fermentation, RSM design of experiments, Optimization

## Abstract

*Lentinus edodes* (*L. edodes*) is one of the most widely used traditional Chinese medicines and a high producer of various bioactive compounds such as polysaccharides. It has been shown that *L. edodes* polysaccharides (LEPLS) have several physiological effects with potential medical applications. In addition, the ability of *L. edodes* to grow and produce bioactive compounds on industrial by-products makes it an excellent candidate for the lage-scale production of such compounds. The objective of this study was to optimize mycelium and polysaccharide production by *L. edodes* on walnut shell through a two-step procedure including a one-factor-at-a-time approach to select the most important factors and a response surface methodology design to determine their optimum combinations. Several factors were evaluated in the first step and among them inoculum size, incubation time, and C/N ratio were selected for optimization of using RSM. The RSM model estimated that a maximal yield of biomass and LEPLS (0.043 mg/g and 46.80 mg/g respectively) could be obtained when inoculum size, incubation time, and C/N ratio were set at 23.41, 30, 10 units, respectively. These values were also verified by validation experiments.

## Introduction

Edible Mushrooms are commonly used for the development of drugs and nutraceuticals (1, 2). Recently, mushrooms have drawn increasing attention due to their potent antioxidant properties (1, 3). *Lentinus edodes* (*L. edodes*) is one of the most widely used traditional Chinese medicines and high producer of various bioactive compounds that is frequently used in Asian countries such as China, Korea, and Japan (4). Among the various bioactive components of *L. edodes*, polysaccharides have been identified as the major bioactive compounds, which are associated with multiple pharmacological effects (3, 5). Indeed, the fungus is an edible mushroom which is attributed not only to its nutritional value but also to the possible potential for production of strong biological active compounds such as total polysaccharides (6). It can be explained by the enormous potential of the *L. edodes* mycelium Polysaccharides (LEPLS), that have been found to be an excellent antioxidant and immuno-modulator substance that could enhance immune responses (1, 3, 5, 7). Hence, total polysaccharides have become an attractive object for modern pharmaceutical research. On the other hand, the production of polysaccharides on the solid substrate is more advantageous for the industry (8, 9). However, the production of the total polysaccharides depends on substrate composition, cultivation system, and environmental conditions affecting fungal growth. Nevertheless, the selection of such substrate is important to interest for polysaccharides production in solid-state fermentation. The selected raw material should be very cheap, and easily available (10, 11). Up to now, various agro-industrial by-products have been used as solid substrate for industrial production of different bioactive compounds (12, 13). According to the latest statistic from the Food and Agriculture Organization of the United Nations, Iran is the second largest producer of walnut in the world with a production of 452,000 tons between 2012 and 2013 (14). Indeed, during the industrial food processing of Walnut shell by-products in Iran, a solid residue known as Walnut shell by-products, which is annually a considerable waste material, is produced. The disposing of this waste can be very expensive, and it may be difficult for producers. These valuable substrates have been mainly formed in lignocellulosic structure which consists of lignin, cellulose, and hemicellulose.

It was the objective of this study to develop potential application for Walnut shell by-products by utilizing them as a substrate for production of valuable compounds. On the other hand, solid-state fermentation (SSF) is a potential technology for application in the production of biologically active compounds. Thus, Walnut shell by-product reuse in this fermentation system due to its desirable characteristics allows obtaining different bioactive compounds, such as total polysaccharides.

Indeed, solid-state fermentation is proposed to convert food-processing wastes such as walnut shell by-products into valuable compounds. In this work, Walnut shell may be a prominent substrate for solid-state fermentation, because the abundant quantity of this material has been discharged by the food industry. Thus, this present study was aimed to evaluate the use of Walnut shell as a solid substrate to cultivate *L. edodes* (strain D.P.B 319) for the improved production of polysaccharides. This work was also addressed on how different conditions along with Walnut shell have affected on *L. edodes* for polysaccharides production under solid-state conditions. Perhaps, in future, our attempt would be helpful for researchers to exploit this organic material as a suitable substrate in industry. 

On the other hand, response surface methodology (RSM), which has been extensively applied in optimization of condition of fermentation, is a powerful and useful method in experimental design that was used to investigate the influence of different variables (15). Thus, the optimal fermentation conditions of *L. edodes* polysaccharide using Walnut shell by-products as a substrate were investigated and RSM design of experiments (DOE) method was used as the statistical method (16-18).

Indeed, the objective of this study was to investigate the influences of different conditions on the potential of *Lentinus edodes* for the production of total polysaccharides. A mathematical model was developed to show the effect of each growth conditions and their interactions on the production of mycelial biomass and polysaccharide (19, 20).

## Experimental

*WSB substrate*

Walnut shell by-product (WSB) was provided by a food company in Iran, where they had been produced as waste products from processing food. Then dried to 2% humidity in an oven and then ground to obtain 2 mm particle size using a standard sieve, collected and packed in polyethylene bags and kept in the dark place at -20 °C until required for analysis.

*Microorganism and media*

*L. edodes* (strain D.P.B 319) was obtained from the Fermentation Laboratory, School of Pharmacy, Shahid Beheshti University, Iran. The stock culture was maintained on malt extract agar (MEA) at 4 °C and was sub-cultured monthly. The cultures were inoculated with mycelia and incubated at 25 °C for 14 days.

*Inoculum preparation and solid-state fermentation*

The stock culture was maintained on malt extract agar (MEA) supplemented with 2% rice bran extract at 4 °C. Wheat grains were used as substrate and culture support for inoculum preparation. The spawn medium was prepared by mixing 60 g of the grain (the grain was washed and soaked in sufficient distilled water for 24 h, then the excess water was removed). The medium was packaged (60 g of wheat grains in 250 mL glass bottle) and autoclaved at 121 °C for 45 min. Then, the substrate was inoculated with 5 agar disks (5 mm diameter). The culture was incubated at 25 °C in the absence of light for a period of 14 days. After preparation of inoculum, the WSB substrate was sterilized and based on different conditions, each flask was filled with 45 g of WSB and inoculated. All batch experiments were carried out in Erlenmeyer flasks (250 mL) with different conditions of fermentation according to the experimental design as follows. All solid culture experiments were incubated at 25 °C. After fermentation, the fungal biomass was dried and ground to obtain the powder, collected and kept in the clean vial at -20 °C until required for analysis. All experiments were performed at least in triplicate.

*Sample preparation*

To analyze the biomass and LEPLs content, all samples (fermented WSB) were freeze-dried using benchtop freeze drier (ALPHA 1-2 / LD Plus - Christ, Germany) and ground using a blender (Asantoossharghmodel1000) and also kept in the freezer (-20 °C) until they were required for analysis. 

*Analytical procedures*

*Determination of biomass*

Determination of fungal biomass was carried out using the determination of ergosterol (21, 22). After dry processing, the crushed powder (500 mg) was extracted with methanol solution. 4 mL of methanol was added to 500 mg of pure mycelium powder. The mixture was vortexed for 1 min and then shaken and finally, incubated overnight at 4 for overnight in a dark place. Then the supernatant was collected by centrifuging at 2767g for 10 min. The supernatant was then filtered through 0.2 µm filter. Final separation of ergosterol from extract was performed by high-performance thin-layer chromatography (HPTLC) and the UV spectrum of ergosterol was recorded from 265 to 300 nm. All experiments were performed at least in triplicate.

*Extraction of LEPLs*

After dry processing, the crushed powder was extracted with boiling water for two hours and the water-soluble LEPLs was precipitated by adding eight volumes of 99.5% ethanol and stored at 4 overnight (23). Then, the precipitated polysaccharide was collected by centrifuging at 2767g for 10 min. The precipitate was dissolved in 10 mL of distilled water. All experiments were performed at least in triplicate.

*Determination of polysaccharide content*

Determination of total polysaccharides was carried out using the phenol-sulfuric acid method (24-26). The color reaction was initiated by mixing 10mL of distilled water was added to 1 mg of total polysaccharides and shaken for 5 min followed by filtration using filter paper (Whatman 2). After 15 min, about 1 mL of the supernatant was used for sugar analysis. To estimate the polysaccharide content in solution, followed by 5mL of concentrated H_2_SO_4_. The mixture was left for 10 minutes and the absorbance was measured at 490_nm_ using a spectrophotometer (SHIMADZU UV-1800, Japan). Polysaccharide content was calculated using a D-glucose (Merck, 1.08337) solution as the standard. The results were expressed as milligram of glucose per gram of the fermented WSB. The experiment was carried out in triplicate.

*Fermentation condition*

The effect of different Conditions including inoculum size, C/N ratio, incubation time, moisture content and pH was studied in this work by using WSB substrate for biomass and LEPLs production. Experimental designs including One-factor-at-a-time and RSM were used to evaluate the effect of variables on biomass and LEPLs production. The influence of different Conditions was examined by replacing appropriate Condition in the solid-state fermentation with WSB.

*One-factor-at-a-time*

In each experiment, one factor was changed, while holding all of the other factors constant. Different inoculum sizes (%), incubation time (days), pH value, C/N ratios, and moisture content (%) were initially investigated by the single-factor experiments. The experimental design with the actual values of the variables is shown in Table 1.

**Table 1:** Values of independent variable for the One-factor-at-a-time experiments

**Symbol code**	**Factors**	**Units**	**Type**	**Experimental values**
A	Inoculum Size	%	Numeric	5 - 20
B	C/N		Numeric	5 - 25
C	Incubation time	Days	Numeric	5 - 45
D	Moisture content	%	Numeric	60 - 75
E	pH		Numeric	5 - 6.5

*Response surface methodology (RSM)*

A Central composite design (CCD) with 3 factors at two levels including three replicates at the center point was used for fitting data on a polynomial model. The experimental design consisted of 20 trials and the value of the dependent response was the mean of three replications. Each independent variable was tested at two levels, high and low, which are denoted by (+) and (-), respectively. The experimental design with the coded and actual values of the variables is shown in Table 2, whereas Table S1 shows the detail of the design matrix. In this study, three factors and their levels were chosen based on the results of one-factor-at-a-time, which are essential for the mycelial growth and LEPLs production of *L. edodes*. The modeling and statistical analysis were performed using Design Expert, version 8.0.5software (Stat-Ease Corporation, USA). The mathematical method describing the relationships between the process responses (the yield of biomass or total polysaccharides) and the fermentation conditions was developed. The yield of biomass or LEPLs by *L. edodes* was multiply regressed with respect to the fermentation conditions by the least squares method as follows:

Y = b_0_+ Σb_i_x_i _+ Σ b_ii_b_i_^2 ^+ Σ b_ij_b_i_b_j                                                                                                             _Equ. 1

Where Y is the predicted response variable (Biomass or LEPLs); b0, bi, bii, and bij are regression coefficients of the model (b0 is the model intercept and bi is the linear coefficient) and xi, xj represent the independent variables (fermentation conditions) in form of coded or real values. The accuracy and general ability of the above polynomial model were evaluated by coefficient of determination (R2). From the regression analysis of the variables, the significant levels at 95% level (*p*#0.05) were considered to have the greater impact on response production (Biomass or LEPLs).

*Statistical Analysis*

Data from the RSM design represents the mean of three independent trials and were used for determining the regression coefficient of the model. Moreover, the results were reported as the mean and standard deviation. The experimental results were means ± standard deviation (SD) of triple determinations. Statistical analysis of the model was performed to evaluate the analysis of variance (ANOVA).

## Results and Discussion

*Screening of the factors affecting biomass and LEPLs production*

*One-factor-at-a-time*

**Figure 1:**
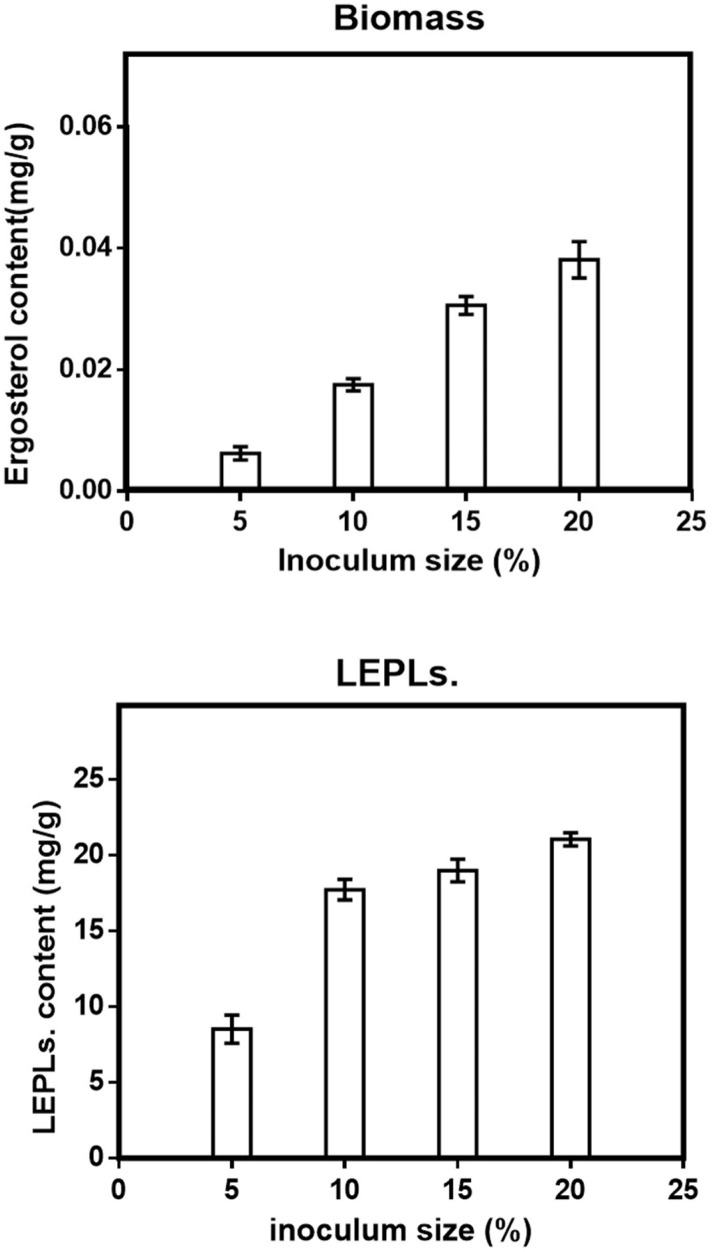
Effect of Inoculum size (a) on the mycelial biomass (b) and LEPLs production in *L. edodes* cultures. The *p*-values (*p *< 0/0001) for significant differences (obtained through one-way ANOVA) are shown

**Figure 2:**
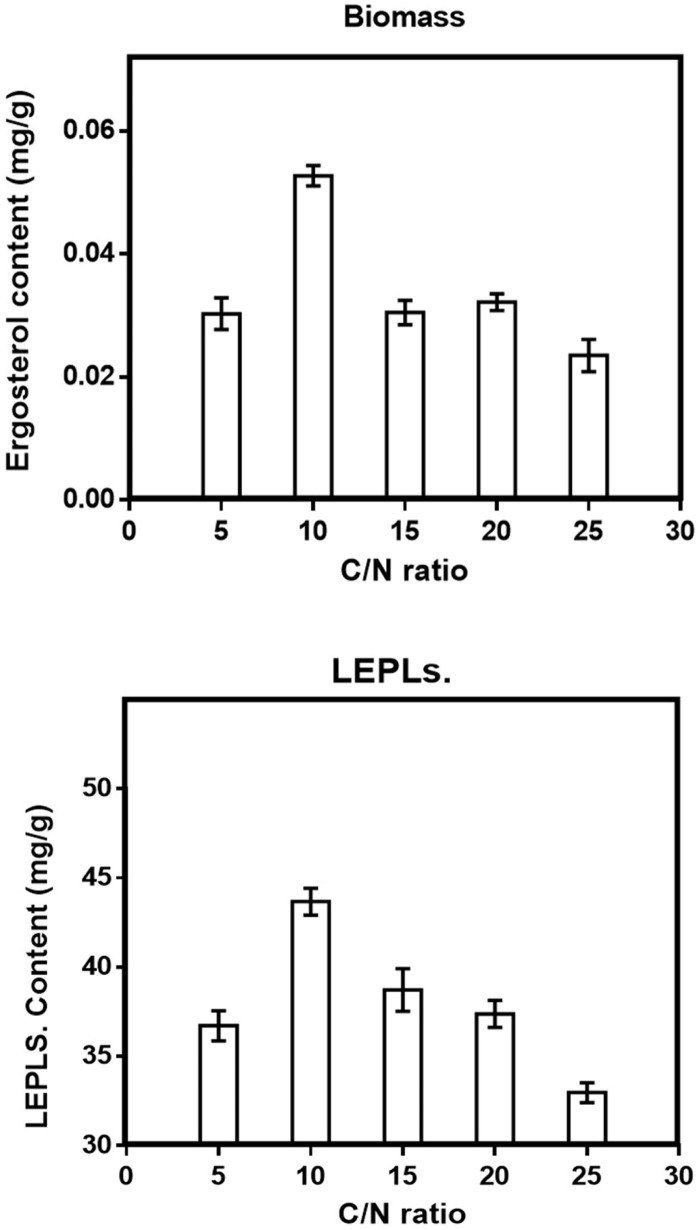
Effect of C/N ratio (a) on the mycelial biomass (b) and LEPLs production in *L. edodes* cultures. The *p*-values (*p *< 0/0001) for significant differences (obtained through one-way ANOVA) are shown

**Figure 3:**
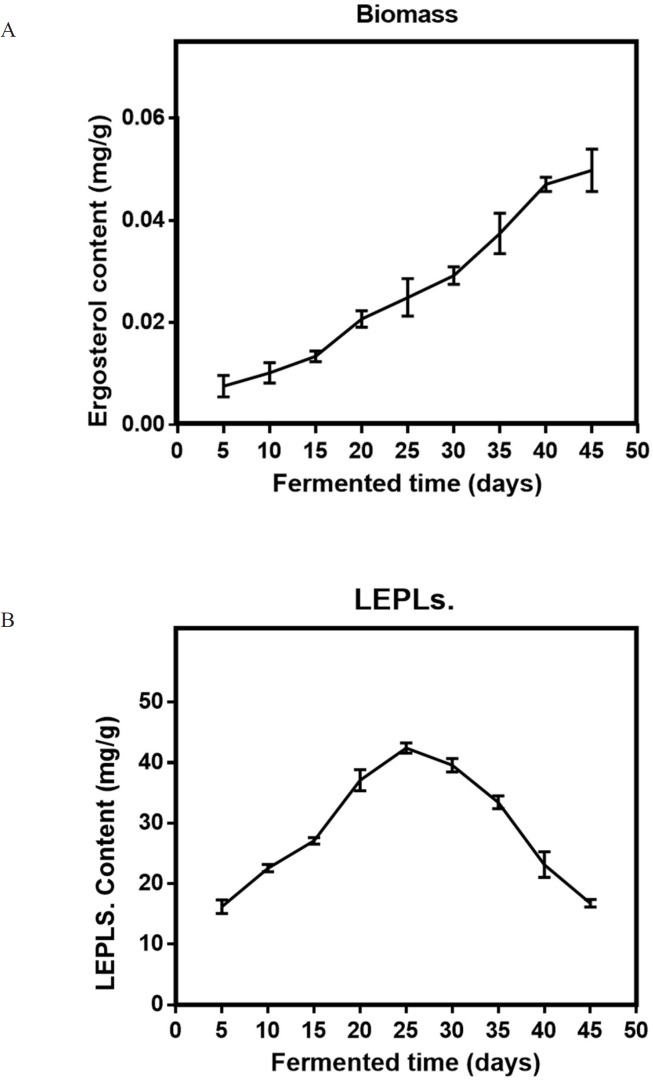
Effect of Incubation time (a) on the mycelial biomass (b) and LEPLs production in *L. edodes* cultures. The *p*-values (*p *< 0/0001) for significant differences (obtained through one-way ANOVA) are shown

**Figure 4:**
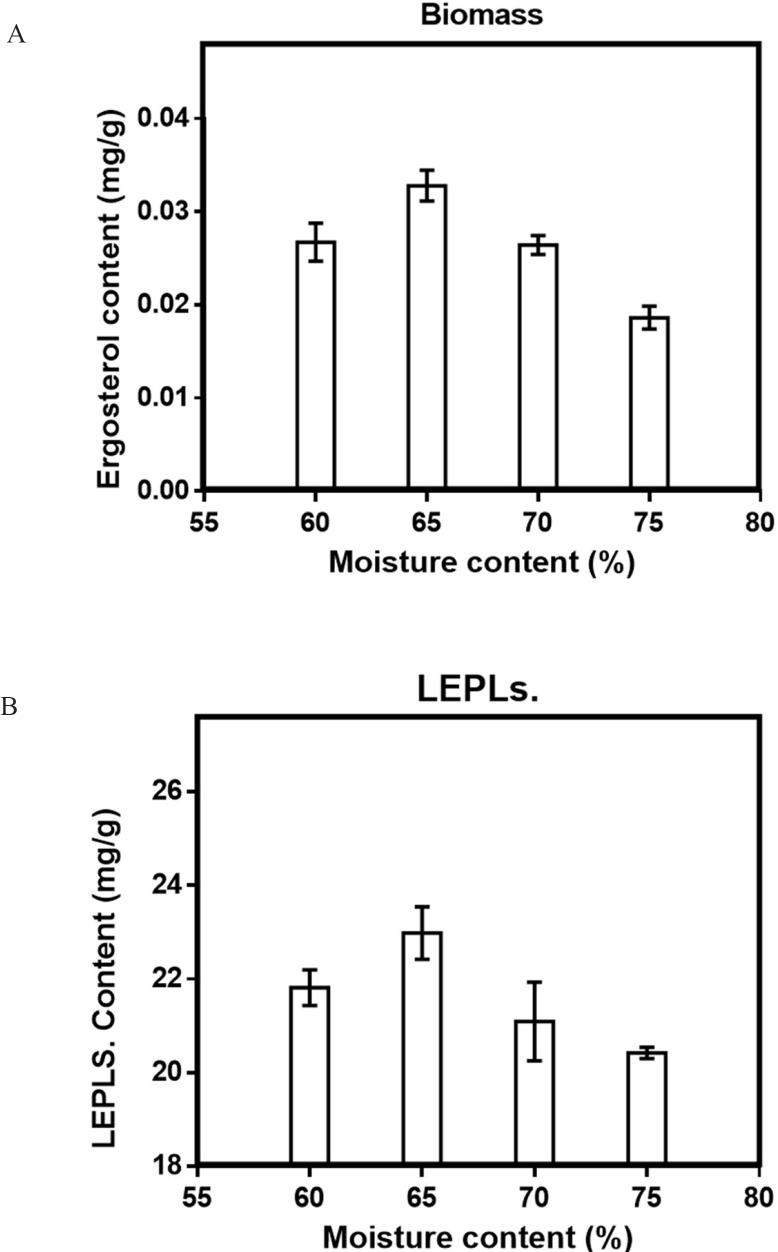
Effect of moisture content (a) on the mycelial biomass (b) and LEPLs production in *L. edodes* cultures. The *p*-values (*p *< 0/0001) for significant differences (obtained through one-way ANOVA) are shown

**Figure 5:**
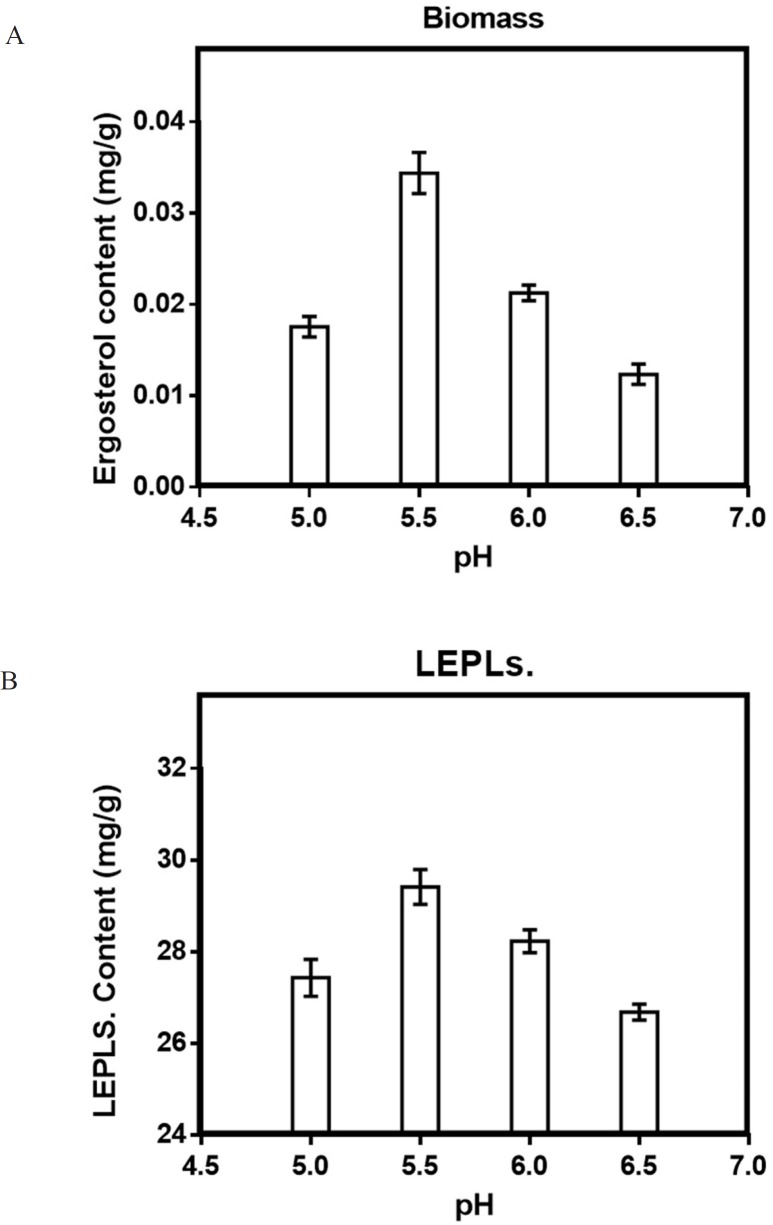
Effect of pH (a) on the mycelial biomass (b) and LEPLs production in *L. edodes* cultures. The *p*-values (*p *< 0/0001) for significant differences (obtained through one-way ANOVA) are shown

Although one-factor-at-a-time methods are tedious, and overlook the interaction between different factors, this method was helpful for the selection of levels in SSF, making the results more reasonable and credible (17). Five variables were chosen in the solid-state fermentation process to efficiently screen out the key factors on the Biomass and LEPLs production (in Table 1). The data reported in Figures 1-5 showed a substantial variation in Biomass and LEPLs production yield among the experimental setting runs, going from 0.033 mg/g to 0.053 mg/g and 21.05 mg/g to 43.67 mg/g, respectively, under different levels of factors, suggesting that the screened parameters were important for the solid-state fermentation of the biomass and LEPLs.

*Effect of inoculum size on solid-state production*

Inoculum size has been reported to play a significant role in the production of biomass and bioactive compounds (27, 28) .

In the present study, in order to determine the actual inoculums size that gave maximal biomass and LEPLs production, fungal spawn was used as the inoculum and different inoculum′s sizes, that is, 5-20 percent (W/W) were studied to enhance the fermentation of substrate and thereby improving biomass and LEPLs production by *L.edodes* in solid-state fermentation (Figure 1). *L.edodes* D.P.B 319 was observed at all the different level of inoculum size studied. When low inoculum level i.e. 5% was used, both of the biomass and LEPLs productions were minimum but as the inoculum level increased, both of two responses were also increased. Maximum yield of biomass and LEPLs was observed at 20% inoculum size (Figure 1, a & b respectively). Statistical analyses were performed by the (ANOVA) with Tukey′s multiple comparisons test (*p *< 0.05). Increased level of inoculum mostly reduced production in the solid-state fermentation process. This may be due to the depletion of nutrients from the fermentation medium which resulted decline in Biomass and LEPLs production. Min Shi *etin*. (2013) reported inoculum size of 12.5% (with a maximum yield of 20.44 mg/g) was best for total polysaccharides production by *Ganoderma lucidum (G. Lucidum)* using Soybean Curd Residue in solid-state fermentation (29). The results indicated 20% of inoculum size was fit for the mycelial growth and enhanced total polysaccharide production. Indeed, there was not much different in biomass and LEPLs production at 20% inoculum size. Thus, it needed to be suitable, when the inoculum size was too small, the fermentation starting time was long. In contrast, a greater inoculum size caused the nutrition to be consumed more quickly, and the fermentation could be interrupted (30-32).

*Effect of C/N ratio on solid-state production*

The C/N ratio is a major factor and an important essential requirement for the growth rate of the mycelium and development of bioactive compounds for the fermentation goals (8). The effect of different ratios of C/N was evaluated for the polysaccharide production by the mushroom.

Based on the total carbon and total nitrogen in WSB, the C/N ratio was calculated (33). C/N ratios of media from 5 to 25 were packed in 250 mL ﬂasks and tested in order to estimate the production of biomass and LEPLs. Sucrose and yeast extracts were used to adjust the C/N ratios.

All of the selected C/N ratios resulted in good mycelial growth and product yield. Among the five different C/N ratios we examined, C/N ratio 10 was the most effective for enhancing the LEPLs production (43.67 mg/g) and biomass (0.053 mg/g) by *L. edodes*. Statistical analyses were performed by the (ANOVA) with Tukey′s multiple comparisons test (*p *< 0.05). However, WSB was low priced and biologically was the most effective energy source, so we chose C/N ratios 10 to be the best C/N ratio for polysaccharides production. Both biomass and LEPLs production increased with increases in C/N ratio from 5 to 10. However, further increases had a negative effect 

(Figure. 2).

*Effect of Incubation time on solid-state production*

A time course of LEPLs production in the solid-state fermentation is presented in Figure 3, b. The result obviously revealed that the yield of the biomass or LEPLs was signiﬁcantly affected by the fermentation time. The biomass production gradually increased with the increasing period of incubation up to 35 days. It was found that highest LEPLs production (42.43 mg/g) was shown at 25 days of fermentation period (Figure 3, b). Statistical analyses were performed by the (ANOVA) with Tukey′s multiple comparisons test (*p *< 0.05). Different experiments were conducted to study the optimum period for maximum LEPLs production in the solid-state fermentation process. Similar observations on maximum total polysaccharides production at 20 days incubation period by *G. lucidum* was reported 43.96 mg/g. Further increase of fermentation period beyond this resulted in a decline in LEPLs production which might be due to the production of toxic metabolites or reduction of nutritional elements during microbial growth which inhibits the mycelial growth or LEPLs production. When TC (2016) studied on total polysaccharides production by *Ganoderma atrum (G. atrum)* and reported that maximum total polysaccharides production was observed in 20 days using wheat as a substrate (34).

*Effect of moisture content on solid-state production*

Although the fermentation with a rang from low initial moisture to very high moisture content has been reported (28, 35), it has been observed that insufficient moisture content led to the decrease in the fungal growth, whereas very high moisture content has an inhibitory effect on biomass and LEPLs production (36-40). To investigate the effect of moisture content on production of biomass and LEPLs by *L. edodes*, WSB substrate was moistened with distilled water from 60-75% (29, 39). The optimal initial moisture for both biomass and LEPLs productions was found to be 65%, (0.033 mg/g and 22.98 mg/g respectively) by *L. edodes*.). Statistical analyses were performed by the (ANOVA) with Tukey′s multiple comparisons test (*p *< 0.05). (Figure 4a, b).

*Effect of pH on solid-state production*

Different pH ranges from 5 to 6.5 was tested to estimate the optimum production of biomass and LEPLs by *L. edodes* in solid-state fermentation. The result was shown in Figure 5. Similar findings were also reported by Min Shi *et al*. (2013) showing optimum pH of 5.5 for polysaccharides production by *G. Lucidum* in basidiomycete culture (29, 41). The study revealed that 5.5 of an initial pH was the optimum pH with 29.4 mg/g of the maximum polysaccharide production. Statistical analyses were performed by the (ANOVA) with Tukey′s multiple comparisons test 

(*p *< 0.05).

*Optimization by RSM design*

Based on the results of the One-factor-at-a-time procedure, three factors (inoculum size, Incubation time, C/N ratio) were selected and RSM was used to optimize the fermentation process. The statistical combinations of critical variables in coded and actual values along with the maximum predicted and experimental responses are listed in Table 2.

**Table 2:** Experimental field definition for the RSM design

**Experimental values**			**Type**	**Units**	**Factors**	**Symbol code**
**High**	**Mean**	**Low**				
20	15	10	Numeric	%	Inoculum Size	A
45	30	5	Numeric		C/N	B
15	10	5	Numeric	Days	Incubation time	C

*Optimization of biomass production by RSM*

Biomass production by *L. edodes* was optimized using central composite design in the Quadratic model, using Design Expert, version 8.0 software (Stat-Ease. Minneapolis, Minn.). Biomass production of this mushroom was optimized by varying the conditions of the fermentation especially the inoculum size, C/N ratio, and the incubation time, at controlled temperature without change in pH during the fermentation. There was a considerable variation in the biomass depending on the fermentation conditions, as shown in Table S1. The replication at the center point conditions resulted in higher biomass than at other levels. Response surface methodology helps in evaluation of the relationship between the dependent variable (biomass yield) and independent variables (fermentation conditions). Observed and predicted values of the biomass yield are shown in Table S1. The accuracy of the model can be seen by the difference between observed and predicted values. 

The statistical significance of Equ. (2) was confirmed by an F-test, and the co-efficient and the analysis of variance (ANOVA) for the response surface quadratic model are presented in Table S2, S3. The fitness of the model expressed using the value of the determination co-efficient (R2). 

The ANOVA of the quadratic regression model demonstrated that the model was significant, with an F-test of a very low probability value (*P *>*F*) <0.0001. In the present study, R2 comes out to be 0.9475 for *L. edodes*. The value of adjusted co-efficient of determination adjusted R2 = 0.9287 for *L. edodes* indicates the high significance of the model. The goodness of the model was indicated by the determination coefficient (R2) and the multiple correlation coefficients (R). The application of RSM yielded the following order polynomial equation.

Y biomass = +0.033 +0.0064A + 0.0021B +0.00235C−0.00453B^2^-0.0051C^2                         ^Equ. 2

Where Y biomass is the response, the logarithmic value of biomass production (mg/g) and also A, B, and C are the uncoded values of the test variables Inoculum size, C/N ratio, and Incubation time. The model coefficients of variation are shown in Figure. S1, S2, and S3- a. the response surface Figures, obtained by the analysis of the experimental data of CCD, indicated a relationship between two variables at a time, while maintaining the third variable at the fixed level. These data are very useful in understanding both linear and interaction effect of the two variables. Figure S1 (a) shows the interaction of Inoculum size and incubation time. As the amount of inoculum size increased between 15 and 20%, at higher incubation period (30 to 45 days), the biomass of *L. edodes* also increased. Figure S2 (a) shows that 10-15 C/N ratio and 15-20% inoculum size gave high biomass yield. Figure S3 (a) shows that biomass yield was maximum, at 30 and 45 days of incubation and C/N ratio 10- 15 was equally effective in increasing the biomass of *L. edodes*. 

The (ANOVA) analysis of biomass production by *L. edodes* showed that *P *> *F* value was 0.0001 and the model was significant for biomass production (response 1) for the *L. edodes*. Also, 

the results indicated that the lack of fit test was insignificant. This result suggested that the sample variation of 94.75% for biomass was attributed to the independent variables, and only about 5.25% of the total variation could not be explained by the Quadratic model. Regression equation (Y Biomass) for the levels of biomass production as functions of Inoculum size (A), C/N ratio (B), and Incubation time (C) suggested that all the three factors influenced biomass production by the *L. edodes*. 

The results predicted the maximum biomass production 0.043 mg/g by *L. edodes* in 1 g of solid walnut shell substrate under the experimental conditions of 23.41% inoculum size, 10 C/N ratio, and 30 days of Incubation time (Table.S1, Run 10). The *P*-value was used to assess the significance of the correlation coefficient; the smaller the value of *P*, the more significant was the corresponding coefficient. As can be seen from results, the coefficients were significant; indicating biomass production depended on all the three factors selected and optimum conditions for maximum biomass production was determined by three-dimensional response surface plots.

*Optimization of LEPLs production by RSM*

LEPLs production by *L. edodes* was optimized using central composite design in the Quadratic model, using Design Expert, version 8.0 software (Stat-Ease. Minneapolis, Minn.). LEPLs production by *L. edodes* mushroom was optimized by varying the conditions of the fermentation especially the inoculum size, C/N ratio, and the incubation time, at controlled temperature without change in pH during fermentation.

There was a considerable variation in the LEPLs depending upon the fermentation conditions, as shown in Table S1. The replication at the center point conditions resulted in higher biomass than at other levels. Response surface methodology helps in evaluation of the relationship between the dependent variable (LEPLs yield) and independent variables (fermentation conditions). The observed and predicted values of the biomass yield are shown in Table S1. The accuracy of the model can be seen by the difference between the observed and predicted values. 

The statistical significance of Eq. (3) was confirmed by an F-test, and the co-efficient and the analysis of variance (ANOVA) for the response surface quadratic model are presented in Table S4, S5. The fitness of the model was expressed using the value of the determination co-efficient (R2). The (ANOVA) of the quadratic regression model demonstrated that the model was significant, with an F-test of a very low probability value (*P* > *F*) <0.0001. In the present study, R2 comes out to be 0.9956 for *L. edodes*. The value of adjusted co-efficient of determination adjusted R2 = 0.9931 for *L. edodes* indicating the high significance of the model. The goodness of the model was indicated by the determination coefficient (R2) and the multiple correlation coefficients (R). The application of RSM yielded the following order polynomial equation.

Y LEPLs = +45.65 +7.47A + 2.79B +5.63C + 1.87AB −4.04A^2^ −5.35B^2^ −7.42C^2…                    ^Equ. 3

Where Y LEPLs is the response, the logarithmic value of LEPLs production (mg/g) and also A, B, and C are the uncoded values of the test variables Inoculum size, C/N ratio, and Incubation time. The model coefficients of variation are shown in Figure. S1, S2, S3- b. Response surface figures, obtained by the analysis of the experimental data of CCD, showed a relationship between two variables at a time, while maintaining the third variable at the fixed level. These Figures are helpful in understanding both linear and interaction effect of the two variables. Figure S1 (b) shows the interaction of Inoculum size and incubation time. As the amount of inoculum size increased between 15 and 20%, at higher incubation period (30 to 45 days), the LEPLs of *L. edodes* also increased. Figure S2 (b) shows that 10-15 C/N ratio and 15-20% inoculum size gave high LEPLs yield. Figure S3 (b) shows that biomass yield was maximum, at 30 and 45 days of incubation and C/N ratio 10- 15 was equally effective in increasing the LEPLs of *L. edodes*. 

The (ANOVA) analysis of LEPLs production by *L. edodes* showed that *P* > F value was 0.0001 and the model was significant for LEPLs production (response 2) for the *L. edodes*. Also, the results indicated that the lack of fit test was insignificant. This result suggested that the sample variation of 99.56% for biomass was attributed to the independent variables, and only about 0.44% of the total variation could not be explained by the Quadratic model. Regression equation (Y LEPLs) for the levels of LEPLs production as functions of Inoculum size (A), C/N ratio (B), and Incubation time (C) suggested that all the three factors influenced LEPLs production by the *L. edodes*. 

The results predicted the maximum LEPLs production 46.80 mg/g by *L. edodes* in 1 g of solid walnut shell substrate under the experimental conditions of 23.41% inoculum size, 10 C/N ratio, and 30 days of Incubation time (Table.S1, Run 10). Similar observations by *G.lucidum* were reported (29). The *P*-value was used to assess the significance of the correlation coefficient; the smaller the value of *P*, the more significant was the corresponding coefficient. As can be seen from the results, the coefficients were significant; indicating LEPLs production depended on all the three factors was selected and the optimum conditions for maximum biomass production were determined by three-dimensional response surface plots.

*Validation of the models*

In order to confirm the predicted results of the model, experiments under optimal conditions were carried out, yielding a biomass of 0.045 mg/g and a LEPLs yield of 47.62 mg/g.

Various substrates using solid-state fermentation technology have been used for the production of polysaccharides by the edible mushrooms (8, 12, 27, 29, 34). In Iran, Walnut shell by-products that are generated from food processing are discarded as waste materials. Based on the literatures, the Walnut shell by-products have many properties that render them suitable substrates for the production of total polysaccharides by solid-state fermentation (14). However, the production of polysaccharides using WSB as a raw material has not been reported. Thus, it was selected as a suitable substrate for total polysaccharides production, which could make full use of the WSB. In the present study, the main factors for the solid-state fermentation conditions of *L. edodes* were scientifically selected for further optimization studies, using an RSM design. In brief, we describe a design process for obtaining the best values and value model using RSM assessments. Through these optimization experiments, highest yield of LEPLs has been 46.91 mg/g (Actual value).

The results from this work indicated that solid-state fermentation of WSB substrate by selected mushroom could be potentially used as an industrial method for total polysaccharides production. 
