# Supplementary material for: Optimization of Growth Conditions of Lentinus edodes Mycelium and Polysaccharides on Walnut Shell by-Products Using Response Surface Analysis
Source: Iran J Pharm Res. 2018 Autumn;17(4):1509–22. (PMC6269588)
Supplement: supplementary-materials: — This PDF file includes:Figures S1 to S3, Tables S1 and S5 [file ijpr-17-1509-s001.pdf]

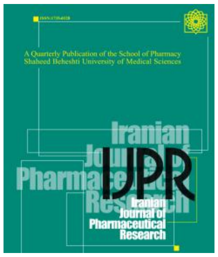

Supplementary Materials for

Optimization of Growth Conditions of *Lentinus edodes* Mycelium and Polysaccharides on Walnut Shell by-products Using Response Surface Analysis

Mahdieh Ameri Shah Reza*, Hossein Vahidi and Farzad Kobarfard

*To whom correspondence should be addressed. E-mail: M.Ameri@sbmu.ac.ir
Volume 17, issue 4 (Autumn 2018**)**

This PDF file includes:

Figures S1 to S3
Tables S1 and S5

a)

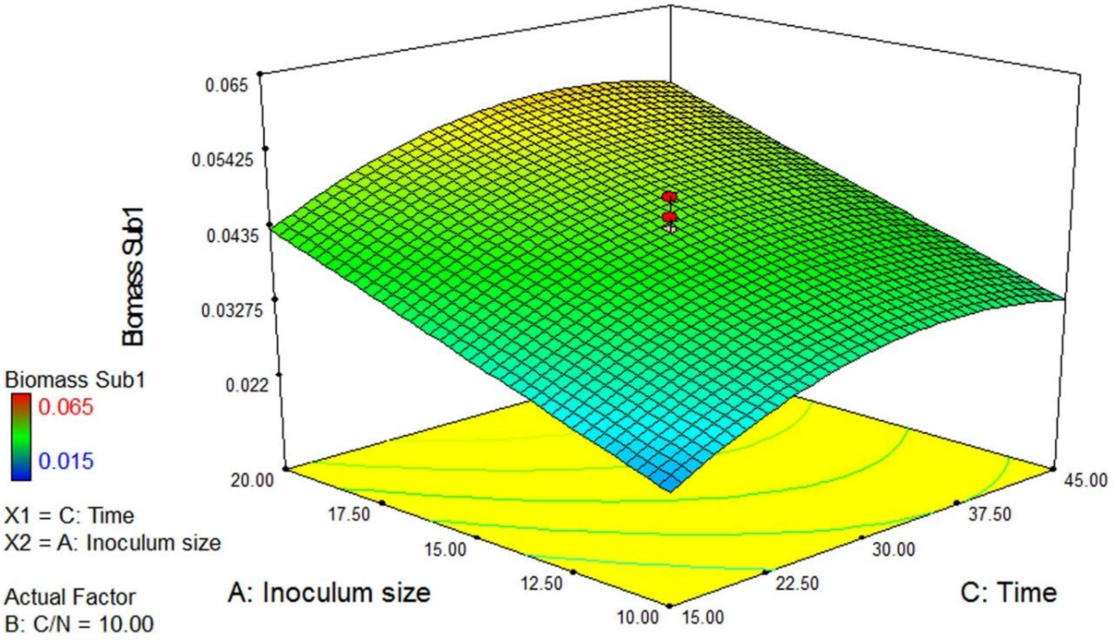

b)

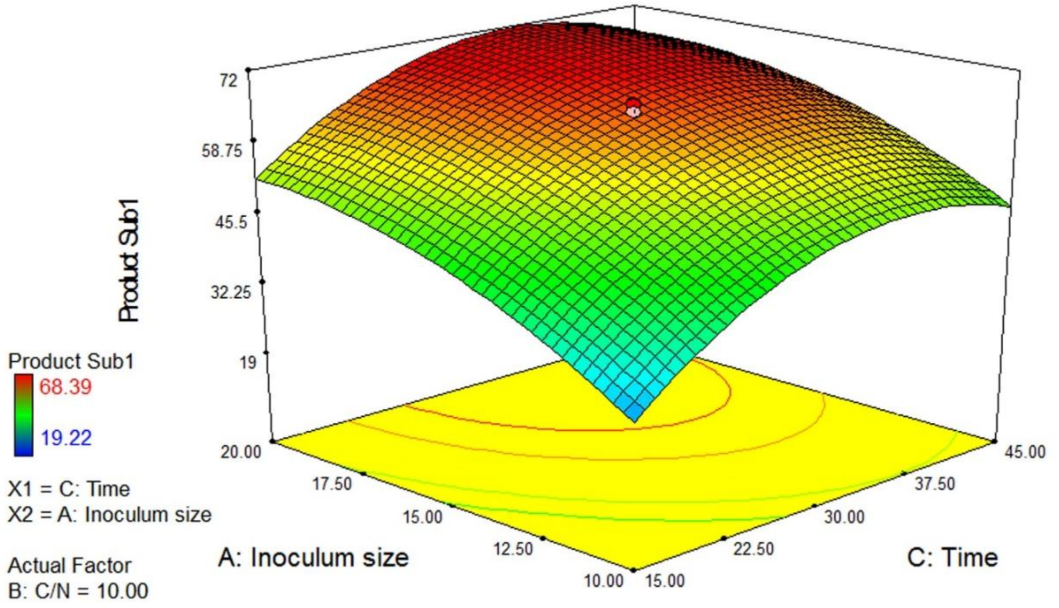

Figure. S1. Response surface plots for LEPLs as a function of fermentation time and inoculum size in *L. edodes* cultures.

a)

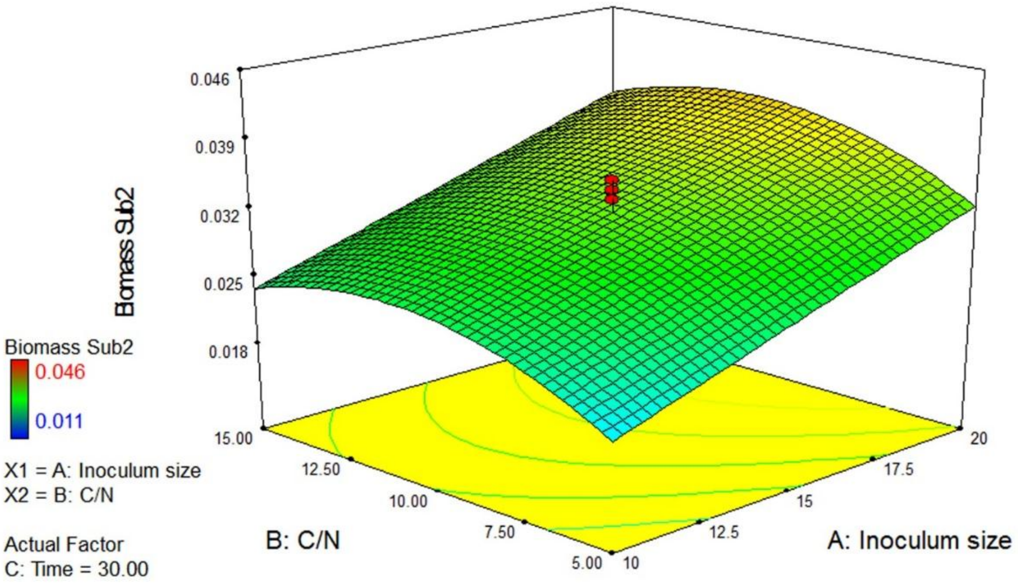

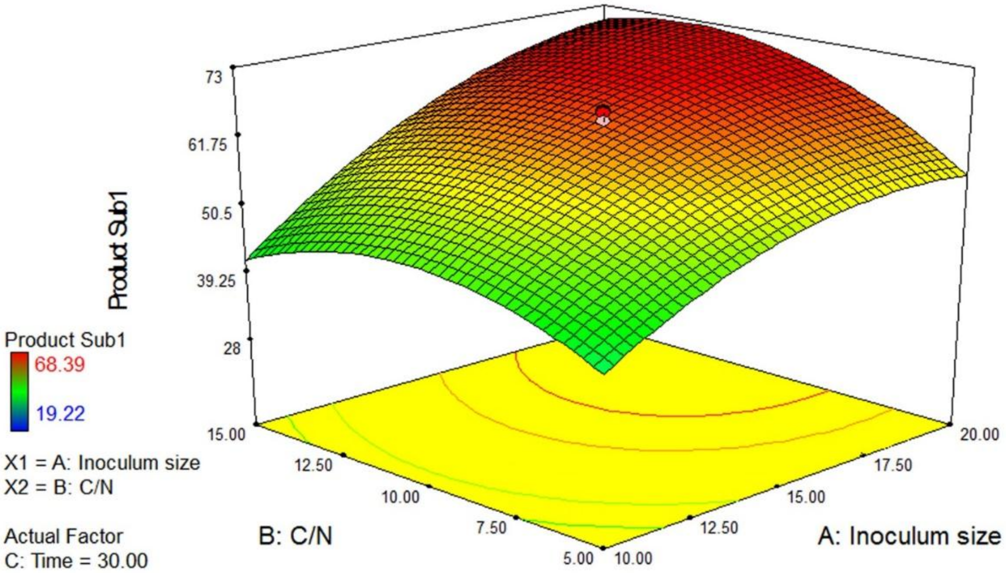

b)

Figure. S2. Response surface plots for LEPLs as a function of inoculum size and C/N ratio in *L. edodes* cultures.

a)

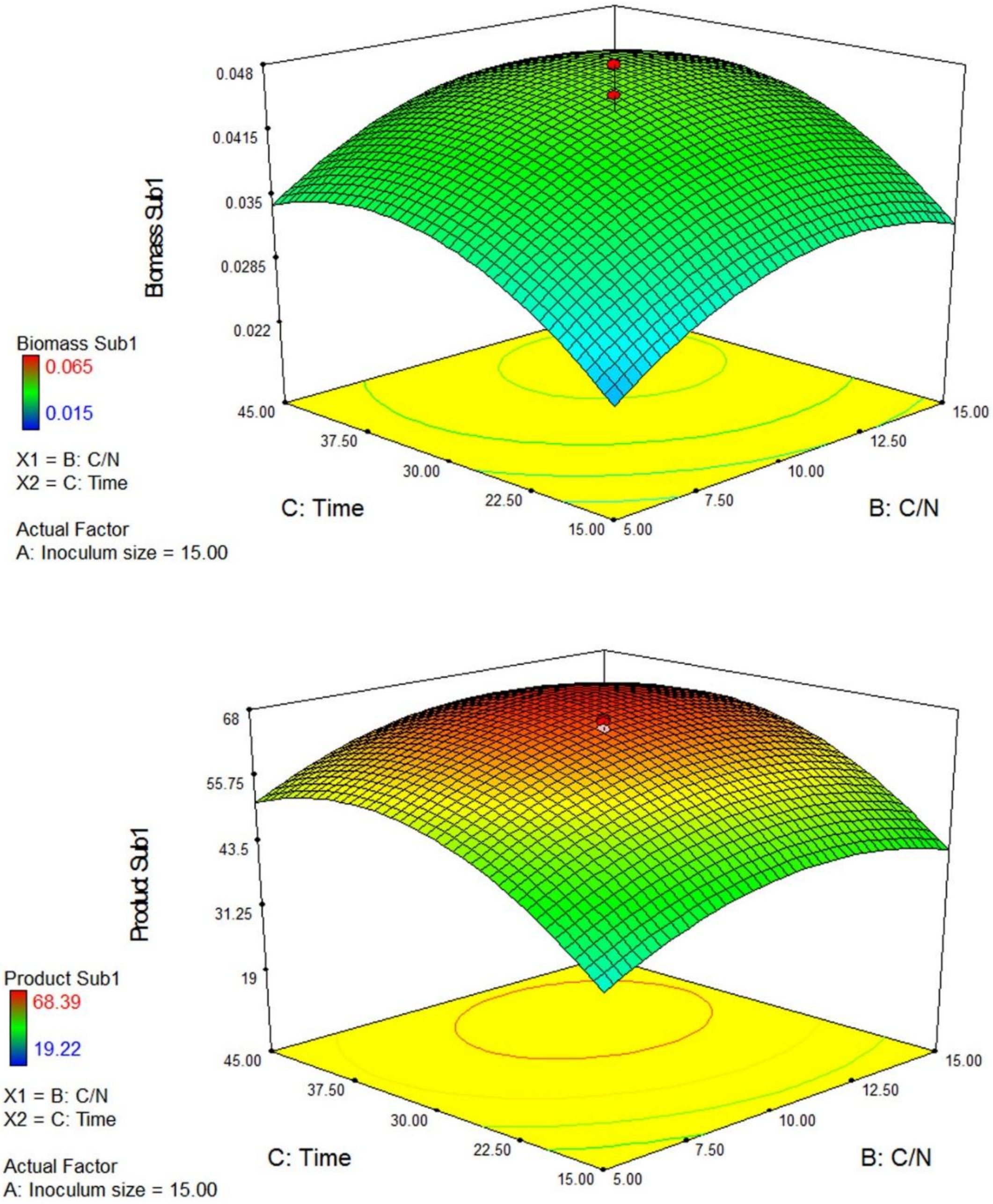

Figure. S3. Response surface plots for LEPLs as a function of fermentation time and C/N ratio in *L. edodes* cultures.

Table S1. CCD design matrix and the response of the dependent variables biomass and LEPLs.

RUN	Factor 1<br>A: Inoculum Size	Factor 2<br>B: C/N	Factor 3<br>C: Incubation time	Actual Value<br>(R1)	Actual Value<br>(R2)
1	10	5	15	0.011	15.38
2	20	5	15	0.022	27.44
3	10	15	15	0.019	16.66
4	20	15	15	0.027	35.11
5	10	5	45	0.019	27.50
6	20	5	45	0.028	36.92
7	10	15	45	0.021	28.10
8	20	15	45	0.033	46.10
9	6.59	10	30	0.018	20.70
10	23.41	10	30	0.046	46.91
11	15	1.59	30	0.018	24.32
12	15	18.41	30	0.023	35.86
13	15	10	4.77	0.016	14.45
14	15	10	55.23	0.022	34.02
15	15	10	30	0.031	45.01
16	15	10	30	0.034	45.07
17	15	10	30	0.034	46.40
18	15	10	30	0.035	46.31
19	15	10	30	0.031	46.11
20	15	10	30	0.033	45.14

Table S2. Results of regression analysis of a full second order polynomial model for optimization of Biomass Production of *L.edodes.*

Source	Std. Dev.	R-Squared	Adjusted R-Squared	Predicted R-Squared	PRESS	
Linear	6.563E-003	0.5003	0.4066	0.2438	1.043E-003	
2FI	7.254E-003	0.5039	0.2750	-0.2894	1.778E-003	
Quadratic	2.274E-003	0.9475	0.9287	0.8838	1.602E-004	Suggested
Cubic	1.629E-003	0.9884	0.9634	0.6769	4.455E-004	Aliased

Table S3. Analysis of variance ANOVA for the fitted linear model for optimization of Biomass Production by *L.edodes*

**Source**	**Sum of Squares**	**df**	**Mean Square**	**F Value**	**p-value Prob> F**
Model	1.307E-003	5	2.613E-004	50.52	**< 0.0001**
A-Inoculum Size	5.554E-004	1	5.554E-004	107.38	**< 0.0001**
B- C/N	5.910E-005	1	5.910E-005	11.43	**0.0045**
	7.541E-005	1	7.541E-005	14.58	**0.0019**
B^2	2.989E-004	1	2.989E-004	57.80	**< 0.0001**
C^2	3.730E-004	1	3.730E-004	72.12	**< 0.0001**
Residual	7.241E-005	14	5.172E-006		
Lack of Fit	5.841E-005	9	6.490E-006	2.32	
Pure Error	1.400E-005	5	2.800E-006		
Cor Total	1.379E-003	19			

Table S4. Results of regression analysis of a full second order polynomial model for optimization of LEPLs. Production of *L.edodes.*

**Source**	**Std. Dev.**	**R-Squared**	**Adjusted R-Squared**	**Predicted R-Squared**	**PRESS**	
Linear	8.89	0.5074	0.4151	0.3200	1744.76	
2FI	9.74	0.5189	0.2968	-0.1410	2927.49	
Quadratic	0.97	0.9956	0.9931	0.9826	44.69	Suggested
Cubic	0.82	0.9984	0.9950	0.8390	413.19	Aliased

Table S5. Analysis of variance ANOVA for the fitted linear model for optimization of LEPLs. Production by *L.edodes.*

**Source**	**Sum of Squares**	**df**	**Mean Square**	**F Value**	**p-value Prob> F**
Model	2554.54	7	364.93	390.32	**< 0.0001**
A- Inoculum Size	761.96	1	761.96	814.96	**< 0.0001**
B- C/N	106.50	1	106.50	113.91	**< 0.0001**
C- Incubation time	433.49	1	433.49	463.64	**< 0.0001**
AB	28.01	1	28.01	29.96	**0.0001**
A^2	234.64	1	234.64	250.96	**< 0.0001**
B^2	412.26	1	412.26	440.93	**< 0.0001**
C^2	793.12	1	793.12	848.29	**< 0.0001**
Residual	11.22	12	0.93		
Lack of Fit	9.01	7	1.29	2.91	**0.1289**
Pure Error	2.21	5	0.44		
Cor Total	2565.76	19			
